# Dentistry’s social contract and dental students’ moral inclusiveness

**DOI:** 10.1186/s12903-023-02994-0

**Published:** 2023-05-10

**Authors:** Astha Shah, Laura Dempster, Sonica Singhal, Carlos Quiñonez

**Affiliations:** 1 Faculty of Dentistry, University of Toronto, Toronto, ON Canada; 2 Public Health Ontario, Toronto, ON Canada; 3 Schulich School of Medicine & Dentistry, University of Western Ontario, London, ON Canada

**Keywords:** Professionalism, Dental Education, Empathy, Social Psychology, Social Justice, Access to Care

## Abstract

**Background:**

Under dentistry’s social contract with the public, dental professionals have a social responsibility to address the oral health needs of the population at large. However, dental education places little emphasis on such moral commitments. By ascertaining dental students’ stance regarding these notions, we may be able to inform changes in dental education. This paper thus explores dental students’ comprehension of dentistry’s social contract using the concepts of moral inclusion, moral community and empathy.

**Methods:**

A cross-sectional online survey collected information from undergraduate dental students at the Faculty of Dentistry, University of Toronto (*N* = 430). Moral inclusion was assessed through the breadth of students’ moral community by computing a “moral inclusion score” (MIS) from Likert scale responses to statements that asked students about their duty of care for different population groups, wherein a higher MIS indicated a broader moral community and in turn greater moral inclusiveness. Empathy was assessed using Likert scale responses to statements that gauged the extent to which students understood the effect of social determinants on people’s health. Association of the MIS with environmental, institutional and student-related factors was also investigated using non-parametric tests and linear regression.

**Results:**

The survey yielded a response rate of 51.4% (*n* = 221). Overall, students in this sample were morally inclusive and displayed empathy. Regression results showed that the MIS was most strongly associated with choosing a small town/rural area as a future practice location (β = 4.76, 95% CI: 0.52, 9.01) and viewing patients as consumers (β = -3.71, 95%CI: -7.13, -0.29).

**Conclusion:**

Students in this sample made morally inclusive choices, which implied that they had a basic understanding of the obligations under dentistry’s social contract. Improving knowledge and experience with regards to addressing the social and economic determinants of oral health and access to oral health care may positively influence students’ perceptions of their professional duties under the social contract.

## Background

Social contract theory emerged with the Greek sophists and gained widespread recognition in the seventeenth and eighteenth centuries through the work of philosophers such as Hobbes, Locke and Rousseau [1]. Simply put, a social contract is an agreement between a group of people living in a society and the society as a whole. As members of the society, the group is required to fulfill the roles and responsibilities that are delineated by their social contract. For learned professions such as dentistry, these responsibilities are embedded in the public good [2]. The dental profession is in a social contract with the public such that in return for acting altruistically and advancing the oral health of the community, the public grants dentists their professional status and rewards [3]. For dentists to maintain their status as professionals, it is imperative that they meet the requirements of their social contract [4].

When it comes to meeting the oral health needs of all members of a society, dentistry’s track-record globally is a cause for concern [5]. In the West, some argue that the private practice model of dentistry has made dental care a commodity that can be accessed only by people for whom it is readily available and affordable, thus impeding access to oral health care for disadvantaged groups [6, 7]. Evidence indicates that many at-risk populations such as low-income individuals, the homeless and Indigenous populations tend to have significantly poorer oral health, greater unmet treatment needs, and more difficulty in accessing care [8–10]. Such inequity can be exacerbated by dental clinics not accepting publicly insured patients, and/or dentists having biased opinions of poverty and/or those who are publicly insured [11, 12].

Such opinions contrast the foundation of the social contract, which calls for the equitable distribution of care and affording substantial care to those who are least advantaged in society [13]. This conceptualization of the social contract also finds mention in the American Dental Education Association (ADEA) values of professionalism in dental education [14]. The values of “Fairness,” which is “demonstrating consistency and even-handedness in dealings with others,” and “Respect,” which is “honoring the worth of others,” embody the essence of a social contract rooted in care for all [14]. While it is expected that graduating dental students understand and embody these values, studies have shown that dental students often hold unfavourable views when asked about their social accountability [15]. They tend to discern that addressing the oral health needs of the poor is a distant issue, and may be unwilling to treat them [16]. Consequently, the dental profession has recognized the need to reform dental education such that future dental professionals understand the responsibilities delineated by their social contract, and work toward removing access barriers and reducing inequity [17].

To inform changes in dental education, it is thus important to ascertain dental students’ stance regarding these and related issues [18]. In this regard, this paper explores dental students’ comprehension of their social contract using the concept of moral inclusion. Moral inclusion is one’s potential to extend moral values and considerations of fairness to others, as well as one’s ability to be conscious of the needs and well-being of all concerned individuals when making decisions [19, 20]. The definition of moral inclusion is not only comparable to dentistry’s social contract [3], it is also aligned with the ADEA values of “Fairness” and “Respect” [14]. Indeed, literature on social justice suggests that perceiving others as equals and respecting them renders a person as morally inclusive [19, 21–23]. Our research endeavour thus aimed to use the idea of moral inclusion to explore dental students’ appreciation of the social contract, with the assumption that a morally inclusive student will exhibit greater fairness, respect and empathy, and, in turn, will be more adept in adhering to the social contract [13, 14].

## Methods

**Fig. 1:**
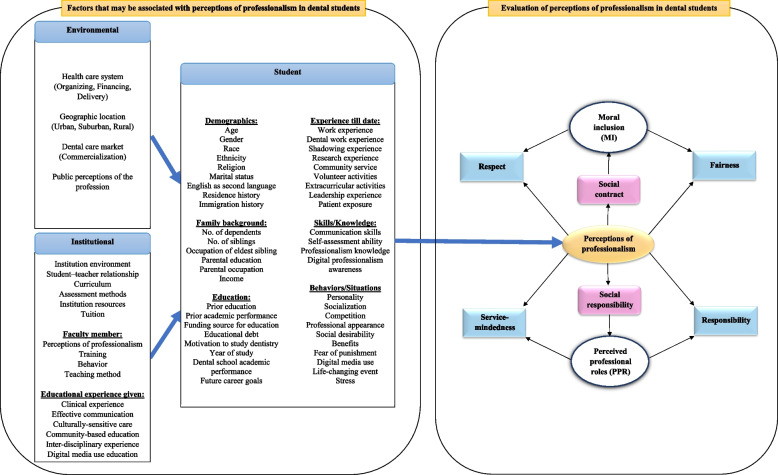
Conceptual framework for perceptions of professionalism in dental students

This paper stems from a larger cross-sectional study conducted at the Faculty of Dentistry, University of Toronto which aimed to evaluate perceptions of professionalism among undergraduate dental students and the factors that may be associated with these perceptions. The conceptual framework presented in Fig. 1 provides the basis for the development and selection of questions for the survey of the larger study [24]. The left side of the framework outlines the environmental, institutional and student-related factors derived from the literature that may affect professionalism in dental students. Factors from this list constitute the exposure variables explored in this paper.

Environmental factors included students’ perceptions on future practice location (geographic location) and their future practice clientele (commercialization; patients vs. consumers), along with whether they had heard cynical views about the dental profession from the public (public perception of the profession). From the institutional factors, we included questions on students’ satisfaction with their curriculum with regards to professionalism education, as well as satisfaction with the teaching of self-assessment techniques, clinical experience, community-based education, culturally sensitive care, interdisciplinary experiences, and effective communication. Additionally, we also inquired about how frequently students had encountered negative experiences with respect to their dental school environment, student–teacher relationships, and in faculty member teaching and behavior. Further, we explored several student-related factors such as personal history and traits, professional and family background and future career aspirations. Questions for the exposure variables were either developed de novo using respective literature sources or adapted from existing questions/instruments (e.g. race/ethnicity) [25]. The right side of the conceptual framework outlines how the ADEA professionalism values have been utilized for assessing professionalism in dental students. As explained in the introduction, this paper addresses the notion of the social contract through the values “Fairness” and “Respect,” and employs the concept of moral inclusion to assess dental students’ ability to apply these values [14]. Dental students’ moral inclusiveness was estimated by determining the breadth of their moral community and their level of empathy.

As mentioned in the introduction, moral inclusion is described by scholars in the field of social psychology as the ability to extend moral values and considerations of fairness to others [19, 20]. Further, the group(s) of people towards whom an individual applies moral inclusion represents their moral community [19]. To assess moral inclusion in dentists, Yu et al. [26, 27] utilized the concept of moral community. As per Yu et al. [26, 27], according to Opotow [19, 28], moral inclusion is generally applied to people who fall within the boundary of one’s moral community, i.e. those people with whom one is strongly connected and to whom one feels morally obligated. These people are referred to as belonging to one’s “in-group” [20]. On the other hand, moral exclusion, which is characterized by processes such as biased evaluation, derogation, dehumanization, and victim blaming, is extended toward people who fall outside one’s moral community, and are seen as a part of one’s “out-group” [19, 20].

Applying this concept, we assessed the moral inclusiveness of students in our study through the range of their moral community. Adapting the methodology used by Yu et al. [26, 27], we inquired about the extent of students’ agreement about their duty of care toward population groups different or distant from themselves (e.g. ‘Population at large,’ ‘All patients in my practice,’ ‘Children from low-income families’—see Table 2). Students were posed with the following 5-point Likert scale question: “As a dentist, to which patient populations do you think you will have a duty of care?” It was postulated that students who strongly agreed that they had a responsibility to care for groups different or distant from themselves had a broad moral community and were hence deemed to be more morally inclusive. Codes ranging from 1 to 5 were assigned for different levels of agreement, such that a higher code represented greater moral inclusiveness with respect to that population group. ‘Strongly disagree’ was coded as 1, ‘Disagree’ as 2, ‘Neutral’ as 3, ‘Agree’ as 4 and ‘Strongly agree’ as 5. Those respondents who provided a response of ‘Not sure’ were excluded from the analysis (*n* = 6).

Next, as per Yu et al. [26, 27], to calculate the breadth of students’ moral community and in turn their moral inclusiveness, a “moral inclusion score” (MIS) was computed. Weights were applied to the different population groups. The group ‘All patients in my practice’ was given a weight of 1, as literature suggests that students tend to feel accountable for patients who will present at their clinic [15]. At-risk population groups such as ‘Children from low-income families,’ ‘Low-income seniors’ and ‘People with disabilities,’ for whom dental students have shown perceptions of reduced responsibility, were given a weight of 2 [29–31]. A weight of 3 was allotted to the group ‘Population at large,’ because it represents the highest level of moral inclusiveness, as well as to the groups that dental students have shown least responsibility toward, specifically ‘Low-income adults,’ ‘Adults on social assistance’ and ‘Indigenous population’ [15, 29]. Lastly, for each population group, the weight was multiplied by the level of agreement, and the resultant scores for all the groups were summed to yield the MIS for each respondent. Two hundred fifteen (215) valid scores were obtained, with a minimum possible score of 19 and a maximum possible score of 95, wherein a higher score implied having a broader moral community and by extension greater moral inclusiveness.

Empathy, an important attribute of moral inclusion, is often interpreted as being sensitive to the position of another [19, 32]. We have used this interpretation to assess the level of empathy shown by students in relation to moral inclusion. Students were asked to what extent they believed that the health of people was a consequence of their financial situation or life choices, with the assumption that students who had more empathy would not place the blame of being unhealthy solely on people’s circumstances but would rather understand the effect of social and economic determinants on people’s health (see Table 4) [33].

The survey, which had 43 questions, was pilot tested by eight dental public health specialty graduate students for ease of completion and face validity. Administrative and ethical approval to implement the survey were obtained from the University of Toronto Office of the Vice-Provost, Students, and the University of Toronto Health Sciences Research Ethics Board (protocol number 38921) respectively. All undergraduate students (*N* = 430) at the faculty were invited to participate in the survey using the online survey tool SurveyMonkey®. An introductory email was first sent to all students by Carlos Quiñonez (CQ), the supervisor of the study, followed by an invitation email by the lead researcher, Astha Shah (AS). Thereafter, three reminder emails (AS) were sent at three days, one week, and two weeks. All the emails contained information pertaining to informed consent, wherein participants were informed that by clicking the link to the survey they were giving their consent to participate in the study. This process was undertaken twice due to difficulty in achieving an adequate response rate. Thus, the fielding of the survey lasted for about two months, from mid-April to mid-June 2020.

The formula used to calculate the sample size was: (Np)(p)(1-p)/[(Np-1)(B/C)^2^ + (p)(1-p)], where ‘Np’ denotes the size of the population, ‘p’ is the proportion of the population expected to choose one of two response categories (most conservative split is 50/50), ‘B’ is the sampling error (3% or 5%), and ‘C’ is the ‘z’ statistic (1.96) associated with the confidence level of 95%. All undergraduate dental students comprise the sample population, thus the number of responses that were required was: 1) For 3% error: (430)(0.5)(0.5)/[(430–1)(0.03/1.96)^2^ + (0.5)(0.5)] = 307; and 2) For 5% error: (430)(0.5)(0.5)/[(430–1)(0.05/1.96)^2^ + (0.5)(0.5)] = 203. As above, based on a concern over the study’s response rate, CQ and AS decided that a minimum sample of 203 responses would be acceptable.

Data coding and analysis was done using IBM® SPSS® Statistics v. 26. All complete survey responses obtained in the larger study (*n* = 221) were used in the data analysis presented in this paper. A negative skewness was noted in the distribution of the MIS and thus attempts were made to normalize it. Since tests of normality (Kolmogorov–Smirnov Test and Shapiro–Wilk Test) revealed no significant differences between the original MIS and the normalized versions, the original score was used for analysis. Bivariate analysis using Mann–Whitney U tests, Kruskal–Wallis tests and Spearman’s correlation were performed to explore the association of the MIS with the exposure variables. Simple linear regression was performed for variables that were significant at *p* < 0.1 at the bivariate level to obtain unstandardized coefficients and to observe the change in the MIS per unit change in the exposure variables. Variables that had significant associations at *p* < 0.05 in simple linear regression were entered simultaneously in multiple regression to determine the variables with the strongest associations with the MIS. It is important to note that the calculation of the MIS and the subsequent regression analysis was done without controlling for any confounders or biases. Additionally, in order to determine the odds of strongly agreeing/agreeing with the empathy statements and having a broad moral community and in turn being more morally inclusive, the MIS was dichotomized at the median. Respondents who scored at or above the median were considered to be more morally inclusive. The unadjusted odds ratios were generated using binary logistic regression.

## Results

Out of the 430 students who were invited to participate, 221 completed the survey, yielding a response rate of 51.4%. While the frequency distributions for all the categorical exposure variables have been provided elsewhere [24], Table 1 provides some data on the characteristics of the sample. The proportion of students who had obtained a masters and/or a professional degree, had three or more years of work experience before entering dental school, and had majored in a non-health related field prior to dental school, was 24.4%, 29.0% and 19.0% respectively. Nearly a third of the survey respondents (31.7%) were in their first year. A little over half the respondents were female (57%) and 67.4% were 24 years of age or older. When asked about their income, roughly 15% of the respondents reported that their, and if applicable, their spouse’s combined annual pre-tax income was $25,000 or more. For their future, 48.9% and 27.6%, respectively, wanted to pursue a career in private practice or a general dentistry residency or internship. The faculty where the survey was conducted provided data on the gender and education level of all undergraduate dental students at the time of the survey. As per this data, 46.6% and 53.4% of the students identified themselves as male and female respectively. Further, prior to entering dental school, 85.2% had a bachelor’s degree and 14.8% had obtained a master’s degree and/or had completed a professional or doctoral program. When comparing this data with the descriptive characteristics of the sample, it was seen that the sample was more equivalent in terms of gender than in terms of prior education level [24].

**Table 1:** Descriptive characteristics of the sample

Variable		n (% total)
**Age**		221
(What is your age?)		
Less than 24 years		72 (32.6)
24 years and older		149 (67.4)
**Gender**		221
(What is your gender?)		
Male		95 (43.0)
Female		126 (57.0)
**Race/ethnicity**		220
(What is your ethnicity/racial background?)		
Others		156 (70.9)
White		64 (29.1)
**Family income**		202
(Approximately, what is your, and if applicable, your spouse’s combined, annual pre-tax income?)		
No income		132 (65.3)
Less than $25,000		40 (19.8)
$25,000 or greater		30 (14.9)
**Prior education level**		221
(Prior to entering dental school, what was your highest level of education?)		
Bachelor’s Degree		167 (75.6)
Master’s Degree/Professional or Doctorate		54 (24.4)
**Prior education field**^**a**^		221
(Which of the following best describes the major fields of study in your previous educational qualifications?)	Major in other fields	47 (21.3)
	Major in biological life sciences	174 (78.7)
		221
	Major in other fields	169 (76.5)
	Major in health-related fields	52 (23.5)
		221
	Major in other fields	179 (81.0)
	Major in non-health related fields	42 (19.0)
**Work experience**		221
(How many years of paid work experience have you had prior to entering dental school?)		
0 or less than 1 year		65 (29.4)
1 – 2 years		58 (26.2)
2 – 3 years		34 (15.4)
More than 3 years		64 (29.0)
**Year of study**		221
(Please indicate your year in the dental program.)		
Year 1		70 (31.7)
Year 2		53 (24.0)
Year 3		45 (20.4)
Year 4		53 (24.0)
**Future career goals**		221
(Please indicate your preferred career path upon graduation.)		
Career in private practice		108 (48.9)
General dentistry residency or internship		61 (27.6)
Others		52 (23.5)

^a^Multiple response categorical exposure variables

The level of agreement with duty of care toward different population groups is outlined in Table 2. The maximum agreement (99.5%) was seen with the population group ‘All patients in my practice,’ while with the maximum disagreement was seen with the groups ‘Population at large’ (4.5%) followed by ‘Indigenous population’ (4.2%). Additionally, 13.3% of respondents had neutral opinions about a duty of care toward ‘Low-income adults,’ while 11.0% and 10.6% respectively held the same opinion for the groups ‘Adults on social assistance’ and ‘People with disabilities.’

**Table 2:** Dental students’ level of agreement with duty of care for different groups

	**n**	**% total**		
		**Strongly Disagree/ Disagree**	**Neutral**	**Agree/ Strongly Agree**
Population at large	220	4.5	8.2	87.2
All patients in my practice	220	0.5	0.0	99.5
Children from low-income families	217	1.4	9.2	89.4
Low-income adults	218	1.8	13.3	84.9
Low-income seniors	218	0.9	9.6	89.5
Adults on social assistance	218	2.3	11.0	86.7
People with disabilities	217	3.7	10.6	85.7
Indigenous populations	216	4.2	10.2	85.7

**Fig. 2:**
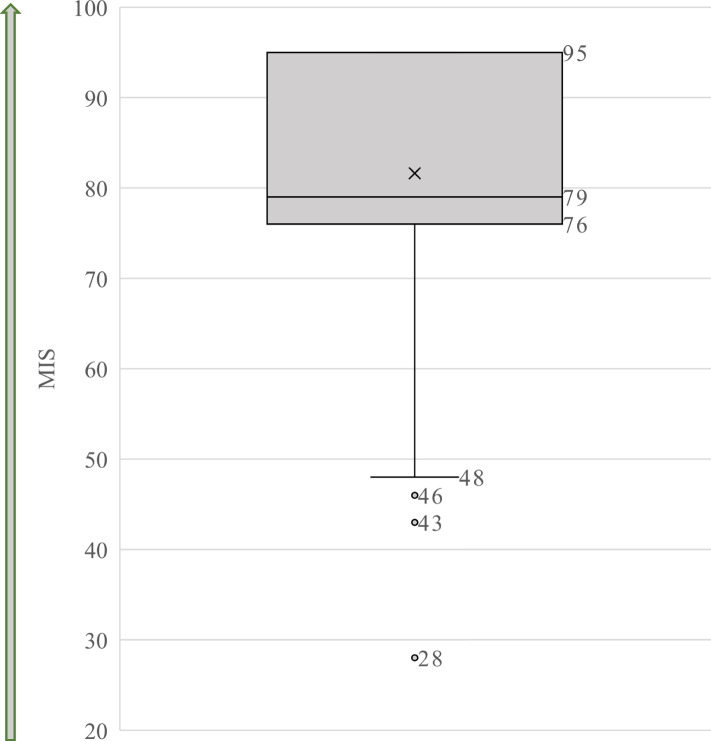
Descriptive characteristics of the moral inclusion score (MIS)

Following the computation of the MIS, a box plot was generated to examine the distribution of the score (Fig. 2). A negative skewness was noted in the distribution of the MIS. The mean MIS was 81.6, the median was 79 and approximately 29% of the respondents provided the maximum score of 95. As noted in the methodology, the MIS score was used, as is, for further analysis, without making any changes to the distribution, since tests of normality revealed no significant differences between the distribution of the original score and the normalized distribution.

Table 3 presents simple linear regression results for exposure variables that were significantly associated with the MIS in bivariate analysis. An increase in the MIS was seen in those students who chose small town/rural areas as their ideal future practice location, whose racial/ethnic background was ‘White,’ who were motivated to study dentistry to become health care professionals, and who had a more conscientious personality. Students who indicated that they were satisfied/very satisfied with the dental school’s teaching methods for developing the ability to self-reflect and for working with other health care professionals also showed a steady rise in the MIS. Similar non-uniform upward trends in the MIS were also observed for students with increasing family income (student and spouse if applicable) and increasing number of years of work experience before starting dental school, as well as with an increase in level of satisfaction with the dental school’s teaching methods for behaving in a professionally responsible manner, for communicating effectively with patients, and for providing dental care to different populations.

**Table 3:** Bivariate and multivariable linear regression of factors associated with the MIS

Variable		Simple linear regression			Multiple regression			
		**Unstandardized coefficients (95% CI)**	**Standard error**	***p*-value**	**Unstandardized coefficients (95% CI)**	**Standard error**	***p*-value**	**Variation Inflation Factor**
Constant (multiple regression)		-	-	-	81.63 (70.36, 92.90)	5.70	< 0.001	-
**Environmental factors**								
**Geographic location** (If you were to own a private practice, in which area would you like to set it up?)								
Urban/Suburban (constant)		80.29 (78.36, 82.19)	0.97	< 0.001	-	-	-	-
Small town/Rural		5.10 (1.37, 8.84)	1.89	0.008	4.76 (0.52, 9.01)	2.15	0.028	1.198
**Dental care market** (Would you perceive the people who come to your future private practice as patients or consumers?)								
Patient (constant)		85.07 (82.80, 87.33)	1.15	< 0.001	-	-	-	-
Consumer		-6.86 (-10.06, -3.66)	1.62	< 0.001	-3.71 (-7.13, -0.29)	1.73	0.034	1.101
**Institutional factors**								
**Institution environment** (Have you seen/experienced a student’s unethical behaviour going unpunished?)								
Never (constant)		85.59 (81.04, 90.14)	2.31	< 0.001	-	-	-	-
Rarely		-3.46 (-8.95, 2.02)	2.78	0.215	-	-	-	-
Sometimes/Often/ Always		-5.87 (-10.99, -0.75)	2.60	0.025	0.73 (-3.14, 4.60)	1.96	0.709	1.380
**Faculty member teaching methods** (Have you seen/experienced a faculty member asking you/another student to instigate unnecessary patient discomfort for students’ learning needs?)								
Never (constant)		83.78 (81.47, 86.09)	1.17	< 0.001	-	-	-	-
Rarely		-4.76 (-8.77, -0.74)	2.04	0.020	-2.49 (-7.15, 2.18)	2.36	0.294	1.647
Sometimes/Often/ Always		-6.02 (-10.58, -1.46)	2.31	0.010	1.83 (-3.99, 7.65)	2.94	0.535	1.976
**Faculty member behaviour** (Have you seen/experienced a faculty member talked about a patient inappropriately to a student or a colleague?)								
Never (constant)		83.65 (81.30, 86.00)	1.19	< 0.001	-	-	-	-
Rarely		-3.31 (-7.15, 0.52)	1.95	0.090	-	-	-	-
Sometimes/Often/ Always		-5.48 (-9.89, -1.08)	2.23	0.015	-1.57 (-6.40, 3.28)	2.45	0.524	1.366
**Curriculum** (Are you satisfied with the dental school’s teaching methods for behaving in a professionally responsible manner?)								
Very dissatisfied/ Dissatisfied/Neutral (constant)		80.56 (77.48, 83.64)	1.56	< 0.001	-	-	-	-
Satisfied		-1.13 (-4.88, 2.62)	1.90	0.554	-	-	-	-
Very Satisfied		9.01 (4.21, 13.81)	2.44	< 0.001	3.61 (-2.41, 9.62)	3.04	0.238	1.950
**Assessment methods** (Are you satisfied with the dental school’s teaching methods for developing the ability to self-reflect?)								
Very dissatisfied/ Dissatisfied/Neutral (constant)		80.89 (78.54, 83.25)	1.20	< 0.001	-	-	-	-
Satisfied		0.22 (-3.26, 3.70)	1.76	0.901	-	-	-	-
Very Satisfied		7.95 (1.96, 13.94)	3.04	0.010	-0.87 (-9.91, 8.17)	4.57	0.849	2.590
**Effective communication** (Are you satisfied with the dental school’s teaching methods for communicating effectively with patients?)								
Very dissatisfied/ Dissatisfied/Neutral (constant)		81.03 (78.24, 83.81)	1.41	< 0.001	-	-	-	-
Satisfied		-1.24 (-4.89, 2.41)	1.85	0.504	-	-	-	-
Very Satisfied		7.52 (2.40, 12.65)	2.60	0.004	6.68 (-1.13, 14.48)	3.95	0.093	2.899
**Culturally sensitive care** (Are you satisfied with the dental school’s teaching methods for providing dental care to different populations?)								
Very dissatisfied/ Dissatisfied/Neutral (constant)		81.15 (78.67, 83.62)	1.25	< 0.001	-	-	-	-
Satisfied		-0.62 (-4.18, 2.94)	1.81	0.733	-	-	-	-
Very Satisfied		5.89 (1.08, 10.69)	2.44	0.017	0.63 (-5.42, 6.69)	3.06	0.837	1.622
**Interdisciplinary experience** (Are you satisfied with the dental school’s teaching methods for working with other health care professionals?)								
Very dissatisfied/ Dissatisfied/Neutral (constant)		79.59 (77.55, 81.63)	1.04	< 0.001	-	-	-	-
Satisfied		3.85 (-0.03, 7.74)	1.97	0.052	-	-	-	-
Very Satisfied		10.41 (4.71, 16.12)	2.89	< 0.001	1.14 (-5.80, 8.08)	3.51	0.746	1.705
**Student factors**								
**Age** (What is your age?)								
Less than 24 years (constant)		83.44 (80.55, 86.32)	1.46	< 0.001	-	-	-	-
24 years and older		-2.71 (-6.24, 0.81)	1.79	0.130	-	-	-	-
**Race/ethnicity** (What is your ethnicity/racial background?)								
Else (constant)		80.60 (78.63, 82.57)	1.00	< 0.001	-	-	-	-
White		3.41 (-0.20, 7.03)	1.83	0.064	-	-	-	-
**Dependents** (How many dependents do you have?)								
None (constant)		81.35 (79.65, 83.05)	0.86	< 0.001	-	-	-	-
1 or more		5.75 (-2.12, 13.62)	3.99	0.151	-	-	-	-
**Family income** (Approximately, what is your, and if applicable, your spouse’s combined, annual pre-tax income?)								
No income (constant)		79.98 (77.87, 82.08)	1.07	< 0.001	-	-	-	-
Less than $25,000		7.02 (2.70, 11.35)	2.19	0.002	2.82 (-1.55, 7.20)	2.21	0.204	1.198
$25,000 or greater		3.67 (-1.32, 8.65)	2.53	0.148	-	-	-	-
**Motivation to study dentistry** (Multiple response variable—Why have you chosen to pursue dentistry?)								
1	Other reasons (constant)	82.30 (80.60, 83.99)	0.86	< 0.001	-	-	-	-
	It’s easy for dentists to find employment	-9.18 (-15.40, -2.95)	3.16	0.004	-5.21 (-11.83, 1.41)	3.35	0.122	1.219
2	Other reasons (constant)	83.13 (81.06, 85.19)	1.05	< 0.001	-	-	-	-
	To have regular work hours when practicing as a dentist	-4.10 (-7.51, -0.69)	1.73	0.019	-2.99 (-6.66, 0.68)	1.86	0.109	1.227
3	Other reasons (constant)	79.56 (77.25, 81.87)	1.17	< 0.001	-	-	-	-
	To become a health care professional	4.18 (0.89, 7.46)	1.67	0.013	2.38 (-1.22, 5.98)	1.82	0.193	1.216
**Year of study** (Please indicate your year in the dental program.)								
Year 1 (constant)		86.37 (83.49, 89.26)	< 0.001	1.47	-	-	-	-
Year 2		-7.20 (-11.55, -2.86)	0.001	2.21	-3.31 (-8.87, 2.24)	2.81	0.240	2.165
Year 3		-5.40 (-10.02, -0.78)	0.022	2.34	-2.95 (-9.33, 3.43)	3.23	0.363	2.602
Year 4		-7.85 (-12.22, -3.49)	< 0.001	2.22	-5.65 (-11.88, 0.58)	3.15	0.075	2.933
**Future career goals** (Please indicate your preferred career path upon graduation.)								
Others (constant)		83.51 (80.05, 86.97)	1.75	< 0.001	-	-	-	-
General dentistry residency		-0.09 (-4.75, 4.57)	2.36	0.968	-	-	-	-
Career in private practice		-3.78 (-7.96, 0.40)	2.12	0.076	-	-	-	-
**Work experience** (How many years of paid work experience have you had prior to entering dental school?)								
0 or less than 1 year (constant)		78.25 (75.24, 81.26)	1.53	< 0.001	-	-	-	-
1 – 2 years		4.89 (0.50, 9.28)	2.23	0.029	2.02 (-2.34, 6.39)	2.21	0.362	1.379
2 – 3 years		3.30 (-1.98, 8.58)	2.68	0.219	-	-	-	-
More than 3 years		5.45 (1.17, 9.73)	2.17	0.013	1.43 (-2.92, 5.79)	2.20	0.517	1.472
**Patient exposure** (Have you seen/experienced a student receiving criticism/verbal abuse from a patient while interacting with them?)								
Never (constant)		88.28 (84.08, 92.48)	2.13	< 0.001	-	-	-	-
Rarely		-7.69 (-13.21, -2.17)	2.80	0.007	-4.08 (-10.30, 2.15)	3.15	0.197	2.668
Sometimes/Often/ Always		-8.44 (-13.14, -3.74)	2.38	< 0.001	-4.37 (-10.81, 2.06)	3.26	0.181	3.755
**Conscientiousness** (Personality)								
Constant		69.51 (60.07, 78.95)	4.79	< 0.001	-	-	-	-
Conscientiousness as a continuous variable		1.59 (0.37, 2.81)	0.62	0.011	0.63 (-0.65, 1.92)	0.65	0.331	1.103

In contrast, students who viewed their future patients as consumers, and who were motivated to pursue dentistry because they believed that it was easy for dentists to find employment and because dentistry had more regular working hours than other health care professions, showed a decline in the MIS. Students who had witnessed unethical student behaviour going unpunished, as well as had seen/experienced faculty members teach and behave unethically showed a consistent downward trend in the MIS. Uniform decreasing trends in the MIS were also observed for students who had chosen a future career in private practice as opposed to other career options, and with increasing frequency of experiencing critical or verbally abusive behaviour from patients. Such trends in reduction of the MIS, although variable, were also noted for students in higher years.

Results of multivariable regression (Table 3) showed that the MIS was most strongly associated with students who chose small town/rural area as an ideal future practice location (small town/rural β = 4.76, 95% CI: 0.52, 9.01) and who viewed future patients as consumers (consumer β = -3.71, 95% CI: -7.13, -0.29). The overall regression model was significant at *p* < 0.001, with R^2^ and adjusted R^2^ values of 0.351 and 0.243 respectively.

Variations were noticed in the levels of agreement with statements that addressed empathy in students (Table 4). While 46.6% disagreed that the poor were less healthy because of their lifestyles, only 10.5% and 14%, respectively, disagreed that the poor were less heathy due to stress/anxiety and that the rich were healthier because they had money to buy healthier things.

**Table 4:** Dental students’ level of agreement with empathy statements

	**n**	**% total**		
		**Strongly Disagree/ Disagree**	**Neither agree nor disagree**	**Agree/ Strongly Agree**
The poor are less healthy because of their lifestyles – they smoke and drink more, don’t exercise and eat junk food	221	46.6	29.4	24.0
The poor are less healthy because they have more stress and anxiety in their lives than those who are better off	221	10.5	17.6	71.9
The rich are healthier because they have money to buy things that make them healthy	221	14.0	17.6	68.3

**Fig. 3:**
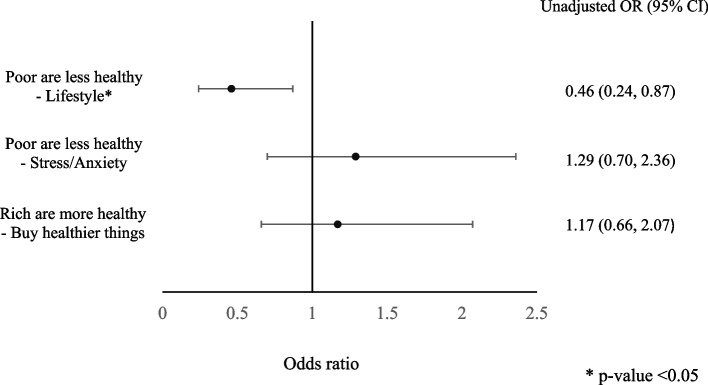
Relationship between agreeing/strongly agreeing with empathy statements and being more morally inclusive

The odds of strongly agreeing/agreeing for each statement and being more morally inclusive (through having a broader moral community) are shown in the forest plot provided in Fig. 3. Respondents who strongly agreed/agreed that the poor are less healthy because of their lifestyles were almost 50% less likely to be morally inclusive than the rest of the respondents.

## Discussion

This study employed the concept of moral inclusion to explore dental students’ understanding of their social contract. Moral inclusion was assessed through the breadth of students’ moral community using duty of care statements and the subsequent calculation of the MIS. It was observed that although students agreed with most statements, they held uncertain opinions about their duty of care for a few population groups. Furthermore, students on the whole had a high MIS, with the choice of future practice location and perception of future patients being most strongly associated with the MIS. Lastly, the relationship between empathy-related statements and the dichotomized MIS suggested that students who believed that the poor were unhealthy because of their lifestyle (as opposed to social and economic circumstance) were less likely to be morally inclusive.

The strong agreement with the duty of care statements and the high average value of the MIS indicate that students report a broad moral community, which is consistent with Yu’s [26, 27] results on the moral community of dentists. This suggests that students in this sample were morally inclusive and hence had a basic understanding of their profession’s social contract. But, similar to practicing dentists, dental students also held some biases [11, 12]. For example, like other studies, this sample of dental students felt more accountable to addressing the oral health needs of patients that would present at their practice as opposed to the needs of the population at large [15]. Furthermore, in accordance with the literature on students’ attitudes toward underserved populations, students in our study showed ambiguity with responsibility toward low-income adults, people with disabilities, and the Indigenous population [29, 31, 34].

The bias in responsibility toward certain groups points to a need to enhance dental students’ comprehension of their obligations under the social contact. The results of the regression analysis and the level of agreement with the empathy statements may give us insight on ways this can be achieved. For example, the multivariable regression analysis revealed that those who choose small/town rural area as an ideal future practice location had a significantly higher MIS. Evidence suggests that, among various reasons, having rural experience can strengthen students’ preparedness and intentions to treat underserved populations [34, 35]. This is also reflected in our simple linear regression results, whereby being ‘very satisfied’ with the dental school’s teaching approach for providing culturally sensitive-care, effectively communicating with patients and working with other health care professionals, resulted in an increasing MIS. One can thus argue that improving dental students’ cultural awareness, communication skills, and community experience may increase their willingness to work with underserved groups, as well as broaden their moral community and augment their understanding of dentistry’s professional obligation to serve diverse populations.

Another factor which was significantly associated with and had a detrimental impact on students’ MIS was viewing future patients as consumers. Some propose that when dentists are commercially driven, they tend to be less altruistic and provide care based on their patients’ ability to pay [36, 37]. In contrast, given that altruism has been shown to have a positive connection with empathy [32], efforts at promoting empathy may result in dental students placing more importance on understanding patients’ needs and circumstances, as well as on improvements in access to care. This set of relationships is seen in our study in the connection between empathy statements and the MIS, where blaming poor patients for their life choices resulted in decreased odds of being morally inclusive, while understanding the role of social and economic determinants in the life of the poor and well-off resulted in increased odds of being morally inclusive. Thus, it may be important for dental schools to explore ways to promote empathy in students, while impressing upon them the importance of social and economic circumstance on determining oral health status and access to oral health care.

While most of the trends in the increase or decrease of the MIS in the regression analysis are consistent with evidence in the literature, there is contrasting and/or insufficient evidence to contextualise the trends for some of the factors associated with the MIS. For example, as opposed to students with some income, students in our sample who had no income had a lesser MIS. Although there is some evidence linking parental income levels to students’ practice plans, further research is needed to determine how students’ own incomes could affect their professional choices [38]. Additionally, students in our sample who identified as ‘White’ tended to have a higher MIS, than their counterparts from other racial/ethnic backgrounds. This contrasts the results of a study conducted in the U.S which found that ‘White’ dental students had greater negative stereotypes about treating Medicaid patients [39].

When interpreting variations related to race/ethnicity, it is important to keep in mind that a bias in responses could have occurred in our sample due to a low response rate. However, limited information about the different characteristics of the entire student population was available at the time of the survey, as the faculty was only able to provide information on gender and prior education level. Thus, the generalizability as well as the potential bias that could exist in our results in terms of characteristics such as race/ethnicity cannot be ascertained. Further, even in our sample population, there was a lack of variation in responses with regards to race/ethnicity exposure variables. While the questions in our survey did attempt to capture students in minority groups, very few responses were recorded for racial/ethnic groups such as Black, Latin American and Arab. Due to this lack of responses, we were unable to ascertain differences which may exist among students belonging to racial and ethnic minorities.

The findings of this study must be viewed in light of some limitations and strengths. For example, gathering self-reported responses may be associated with issues of social desirability and non-response bias. Further, our results are based on a sample of students from only one dental faculty and thus are not representative of dental students in other dental faculties in the same country as well as elsewhere in the world. Moreover, research on assessing moral community and moral inclusion is scarce in the dental literature, which raises concerns regarding the validity and reliability of the questions employed to evaluate moral inclusiveness in this paper. In terms of strengths, this study may be the first of its kind to address dental students’ notions of the social contract through the concepts of moral inclusion and moral community, and is definitely the first to do so in Canadian dental students. Additionally, data analysis was done such that we were able to observe the direction of effect that the exposure variables had on moral inclusion, which can allow educational leaders to identify and incorporate these variables’ concepts when attempting to positively influence dental students’ moral inclusiveness and professionalism.

## Conclusion

Our results suggest that overall dental students had a broad moral community and thus were morally inclusive. The factors most strongly associated with students’ moral inclusiveness were choice of future practice location and perceptions of future patients. Those who had a broad moral community also displayed greater understanding and empathy with respect to the social and economic determinants affecting people’s health. We conclude that although dental students may understand the obligations that delineate their social contract, there is much room for improvement.

## Data Availability

The data that support the findings of this paper are available upon reasonable request from the corresponding author. The data are not publicly available due to privacy and ethical restrictions.

## Abbreviations

ADEA: American Dental Education Association
MIS: Moral Inclusion Score
