# Correction: Dentistry’s social contract and dental students’ moral inclusiveness

**DOI:** 10.1186/s12903-023-03098-5

**Published:** 2023-06-13

**Authors:** Astha Shah, Laura Dempster, Sonica Singhal, Carlos Quiñonez

**Affiliations:** 1 Faculty of Dentistry, University of Toronto, Toronto, ON Canada; 2 Public Health Ontario, Toronto, ON Canada; 3 Schulich School of Medicine & Dentistry, University of Western Ontario, London, ON Canada

**Correction: BMC Oral Health 23, 271 (2023)**

**https://doi.org/10.1186/s12903-023-02994-0**

In Table 3 of this article [1], under the heading “Year of study” the data values for the columns “Standard error” and “*p*-value” in the simple linear regression section have been interchanged.

The corrected Table 3 is given below.

**Table 3:** Bivariate and multivariable linear regression of factors associated with the MIS

Variable		Simple linear regression			Multiple regression			
		**Unstandardized coefficients (95% CI)**	**Standard error**	***p*-value**	**Unstandardized coefficients (95% CI)**	**Standard error**	***p*-value**	**Variation Inflation Factor**
Constant (multiple regression)		-	-	-	81.63 (70.36, 92.90)	5.70	< 0.001	-
**Environmental factors**								
**Geographic location** (If you were to own a private practice, in which area would you like to set it up?)								
Urban/Suburban (constant)		80.29 (78.36, 82.19)	0.97	< 0.001	-	-	-	-
Small town/Rural		5.10 (1.37, 8.84)	1.89	0.008	4.76 (0.52, 9.01)	2.15	0.028	1.198
**Dental care market** (Would you perceive the people who come to your future private practice as patients or consumers?)								
Patient (constant)		85.07 (82.80, 87.33)	1.15	< 0.001	-	-	-	-
Consumer		-6.86 (-10.06, -3.66)	1.62	< 0.001	-3.71 (-7.13, -0.29)	1.73	0.034	1.101
**Institutional factors**								
**Institution environment** (Have you seen/experienced a student’s unethical behaviour going unpunished?)								
Never (constant)		85.59 (81.04, 90.14)	2.31	< 0.001	-	-	-	-
Rarely		-3.46 (-8.95, 2.02)	2.78	0.215	-	-	-	-
Sometimes/Often/Always		-5.87 (-10.99, -0.75)	2.60	0.025	0.73 (-3.14, 4.60)	1.96	0.709	1.380
**Faculty member teaching methods** (Have you seen/experienced a faculty member asking you/another student to instigate unnecessary patient discomfort for students’ learning needs?)								
Never (constant)		83.78 (81.47, 86.09)	1.17	< 0.001	-	-	-	-
Rarely		-4.76 (-8.77, -0.74)	2.04	0.020	-2.49 (-7.15, 2.18)	2.36	0.294	1.647
Sometimes/Often/Always		-6.02 (-10.58, -1.46)	2.31	0.010	1.83 (-3.99, 7.65)	2.94	0.535	1.976
**Faculty member behaviour** (Have you seen/experienced a faculty member talked about a patient inappropriately to a student or a colleague?)								
Never (constant)		83.65 (81.30, 86.00)	1.19	< 0.001	-	-	-	-
Rarely		-3.31 (-7.15, 0.52)	1.95	0.090	-	-	-	-
Sometimes/Often/Always		-5.48 (-9.89, -1.08)	2.23	0.015	-1.57 (-6.40, 3.28)	2.45	0.524	1.366
**Curriculum** (Are you satisfied with the dental school’s teaching methods for behaving in a professionally responsible manner?)								
Very dissatisfied/Dissatisfied/Neutral (constant)		80.56 (77.48, 83.64)	1.56	< 0.001	-	-	-	-
Satisfied		-1.13 (-4.88, 2.62)	1.90	0.554	-	-	-	-
Very Satisfied		9.01 (4.21, 13.81)	2.44	< 0.001	3.61 (-2.41, 9.62)	3.04	0.238	1.950
**Assessment methods ** (Are you satisfied with the dental school’s teaching methods for developing the ability to self-reflect?)								
Very dissatisfied/Dissatisfied/Neutral (constant)		80.89 (78.54, 83.25)	1.20	< 0.001	-	-	-	-
Satisfied		0.22 (-3.26, 3.70)	1.76	0.901	-	-	-	-
Very Satisfied		7.95 (1.96, 13.94)	3.04	0.010	-0.87 (-9.91, 8.17)	4.57	0.849	2.590
**Effective communication ** (Are you satisfied with the dental school’s teaching methods for communicating effectively with patients?)								
Very dissatisfied/Dissatisfied/Neutral (constant)		81.03 (78.24, 83.81)	1.41	< 0.001	-	-	-	-
Satisfied		-1.24 (-4.89, 2.41)	1.85	0.504	-	-	-	-
Very Satisfied		7.52 (2.40, 12.65)	2.60	0.004	6.68 (-1.13, 14.48)	3.95	0.093	2.899
**Culturally sensitive care** (Are you satisfied with the dental school’s teaching methods for providing dental care to different populations?)								
Very dissatisfied/Dissatisfied/Neutral (constant)		81.15 (78.67, 83.62)	1.25	< 0.001	-	-	-	-
Satisfied		-0.62 (-4.18, 2.94)	1.81	0.733	-	-	-	-
Very Satisfied		5.89 (1.08, 10.69)	2.44	0.017	0.63 (-5.42, 6.69)	3.06	0.837	1.622
**Interdisciplinary experience** (Are you satisfied with the dental school’s teaching methods for working with other health care professionals?)								
Very dissatisfied/Dissatisfied/Neutral (constant)		79.59 (77.55, 81.63)	1.04	< 0.001	-	-	-	-
Satisfied		3.85 (-0.03, 7.74)	1.97	0.052	-	-	-	-
Very Satisfied		10.41 (4.71, 16.12)	2.89	< 0.001	1.14 (-5.80, 8.08)	3.51	0.746	1.705
**Student factors**								
**Age** (What is your age?)								
Less than 24 years (constant)		83.44 (80.55, 86.32)	1.46	< 0.001	-	-	-	-
24 years and older		-2.71 (-6.24, 0.81)	1.79	0.130	-	-	-	-
**Race/ethnicity** (What is your ethnicity/racial background?)								
Else (constant)		80.60 (78.63, 82.57)	1.00	< 0.001	-	-	-	-
White		3.41 (-0.20, 7.03)	1.83	0.064	-	-	-	-
**Dependents** (How many dependents do you have?)								
None (constant)		81.35 (79.65, 83.05)	0.86	< 0.001	-	-	-	-
1 or more		5.75 (-2.12, 13.62)	3.99	0.151	-	-	-	-
**Family income** (Approximately, what is your, and if applicable, your spouse’s combined, annual pre-tax income?)								
No income (constant)		79.98 (77.87, 82.08)	1.07	< 0.001	-	-	-	-
Less than $25,000		7.02 (2.70, 11.35)	2.19	0.002	2.82 (-1.55, 7.20)	2.21	0.204	1.198
$25,000 or greater		3.67 (-1.32, 8.65)	2.53	0.148	-	-	-	-
**Motivation to study dentistry** (Multiple response variable—Why have you chosen to pursue dentistry?)								
1	Other reasons (constant)	82.30 (80.60, 83.99)	0.86	< 0.001	-	-	-	-
	It’s easy for dentists to find employment	-9.18 (-15.40, -2.95)	3.16	0.004	-5.21 (-11.83, 1.41)	3.35	0.122	1.219
2	Other reasons (constant)	83.13 (81.06, 85.19)	1.05	< 0.001	-	-	-	-
	To have regular work hours when practicing as a dentist	-4.10 (-7.51, -0.69)	1.73	0.019	-2.99 (-6.66, 0.68)	1.86	0.109	1.227
3	Other reasons (constant)	79.56 (77.25, 81.87)	1.17	< 0.001	-	-	-	-
	To become a health care professional	4.18 (0.89, 7.46)	1.67	0.013	2.38 (-1.22, 5.98)	1.82	0.193	1.216
**Year of study** (Please indicate your year in the dental program.)								
Year 1 (constant)		86.37 (83.49, 89.26)	1.47	< 0.001	-	-	-	-
Year 2		-7.20 (-11.55, -2.86)	2.21	0.001	-3.31 (-8.87, 2.24)	2.81	0.240	2.165
Year 3		-5.40 (-10.02, -0.78)	2.34	0.022	-2.95 (-9.33, 3.43)	3.23	0.363	2.602
Year 4		-7.85 (-12.22, -3.49)	2.22	< 0.001	-5.65 (-11.88, 0.58)	3.15	0.075	2.933
**Future career goals** (Please indicate your preferred career path upon graduation.)								
Others (constant)		83.51 (80.05, 86.97)	1.75	< 0.001	-	-	-	-
General dentistry residency		-0.09 (-4.75, 4.57)	2.36	0.968	-	-	-	-
Career in private practice		-3.78 (-7.96, 0.40)	2.12	0.076	-	-	-	-
**Work experience** (How many years of paid work experience have you had prior to entering dental school?)								
0 or less than 1 year (constant)		78.25 (75.24, 81.26)	1.53	< 0.001	-	-	-	-
1 – 2 years		4.89 (0.50, 9.28)	2.23	0.029	2.02 (-2.34, 6.39)	2.21	0.362	1.379
2 – 3 years		3.30 (-1.98, 8.58)	2.68	0.219	-	-	-	-
More than 3 years		5.45 (1.17, 9.73)	2.17	0.013	1.43 (-2.92, 5.79)	2.20	0.517	1.472
**Patient exposure** (Have you seen/experienced a student receiving criticism/verbal abuse from a patient while interacting with them?)								
Never (constant)		88.28 (84.08, 92.48)	2.13	< 0.001	-	-	-	-
Rarely		-7.69 (-13.21, -2.17)	2.80	0.007	-4.08 (-10.30, 2.15)	3.15	0.197	2.668
Sometimes/Often/Always		-8.44 (-13.14, -3.74)	2.38	< 0.001	-4.37 (-10.81, 2.06)	3.26	0.181	3.755
**Conscientiousness** (Personality)								
Constant		69.51 (60.07, 78.95)	4.79	< 0.001	-	-	-	-
Conscientiousness as a continuous variable		1.59 (0.37, 2.81)	0.62	0.011	0.63 (-0.65, 1.92)	0.65	0.331	1.103
